# Analysis of patient-directed search content and online resource quality for ulnar collateral ligament injury and surgery

**DOI:** 10.1016/j.jseint.2023.11.017

**Published:** 2023-12-14

**Authors:** Johnathon R. McCormick, William E. Harkin, Alexander J. Hodakowski, John T. Streepy, Zeeshan A. Khan, Colton C. Mowers, Braedon R. Urie, Harkirat S. Jawanda, Garrett R. Jackson, Jorge Chahla, Grant E. Garrigues, Nikhil N. Verma

**Affiliations:** Rush University Medical Center, Department of Orthopedic Surgery, Chicago, IL, USA

**Keywords:** Ulnar collateral ligament tear, Ulnar collateral ligament surgery, Google, People also ask, Online questions, Patient education

## Abstract

**Background:**

Patients use the Internet to learn information about injuries, yet online content remains largely unstudied. This study analyzed patient questions posed online regarding ulnar collateral ligament (UCL) tears or UCL surgical management.

**Methods:**

Three separate search strings about UCL tear and UCL surgery were queried on the Google search engine. The 300 most commonly asked questions were compiled for each topic and associated webpage information was collected from the “People also ask” section. Questions were categorized using the Rothwell classification and webpages by *Journal of the American Medical Association* (JAMA) benchmark criteria.

**Results:**

The most frequent UCL tear questions were “how long does it take to heal a torn UCL?” and “what is nonsurgical treatment for the UCL?” The most frequent UCL surgery question was “can you retear your UCL after surgery?” The Rothwell classification of questions for UCL tear/UCL surgery was 55%/32% policy, 38%/57% fact, and 7%/11% value with highest subcategories being indications/management (46%/25%) and technical details (24%/25%). The most common webpages were academic (39%/29%) and medical practice (24%/26%). Mean JAMA score for all 600 webpages was low (1.2), with journals (mean = 3.4) having the highest score. Medical practice (mean = 0.5) and legal websites (mean = 0.0) had the lowest JAMA scores. Only 30% of webpages provided UCL-specific information.

**Conclusion:**

Online UCL patient questions commonly pertain to technical details and injury management. Webpages suggested by search engines contain information specific to UCL tears and surgery only one-third of the time. The quality of most webpages provided to patients is poor, with minimal source transparency.

The current era of digital health allows patients to query any number of online search engines regarding their musculoskeletal injuries and instantaneously access a myriad of unfiltered resources. A prior Pew Research Center survey study found 90% of Americans used the Internet at least once in the past year and 72% of that group searched the Internet for answers to health-related questions.^7^ While accessibility of information remains a positive of the Internet age, the accuracy, reliability, and quality of the web pages that patients may find is unsubstantiated and of low academic quality. Websites related to musculoskeletal pathology and procedures provided by search engines have previously been shown to be unreliable and of low quality for multiple orthopedic issues.^9^^,^^15^ The current healthcare landscape relies on a shared decision-making model between the patient and physician—one that can become strained if patients rely on incomplete or false online information which is in disagreement with their surgeon’s recommendations. It is imperative that physicians understand the information their patients seek out so as foster more productive conversations when discussing future treatment.

Ulnar collateral ligament (UCL) injury occurs when the ligament is put under extreme stress or through cumulative microtrauma that can cause the ligament to attenuate and rupture. Injury to the UCL is seen disproportionately in young overhead throwing athletes, and it has been estimated that approximately 25% of Major League Baseball pitchers undergo reconstruction for UCL injury at some point in their career.^6^ Misinformation is common with this relatively infrequent injury, with 51% of high school players and 37% of parents believing that UCL surgery should be performed on players without injury to enhance performance.^1^ It has been well established that patients with appropriate preoperative education have a reduction in postoperative complications and adverse events.^2^^,^^8^^,^^11^ To date, patient questions pertaining to UCL injury and UCL surgery have been limited to surveys, without analyzing the countless online searches performed each and every day. Recently, Google has incorporated a “People also ask” tool which uses machine learning to suggest additional questions and resources based on data gathered from previous searches. Each “People also ask” question includes a snippet of text aiming to answer the original question, with a link to the webpage with the information.

The purpose of the present study was to (1) investigate the most frequent questions patients search online regarding both UCL tears and UCL surgery and (2) determine the type and quality of webpages provided to patients from the webpage results to each query.

## Methods

### Data collection

We used the methods of Shen et al, which have previously been applied to upper extremity queries as well.^9^^,^^13^^,^^15^ Briefly, this study was conducted using publicly available, de-identified data to protect individual privacy, and as such, did not require Institutional Review Board approval or informed consent. On February 26, 2023, a publicly available Google Chrome extension SEO Minion (Axeman Technology Solutions, LLP, Mumbai, India) was used to perform a query in Google Search pertaining to the following 2 topics that a patient may be expected to research online: UCL injuries and UCL surgery. Three search strings were independently entered into the search bar for the UCL tear group: “elbow ulnar collateral ligament injury”, “elbow ulnar collateral ligament tear”, and “elbow ulnar collateral ligament rupture”. The 3 strings used for the UCL surgery group were “elbow ulnar collateral ligament reconstruction”, “elbow ulnar collateral ligament surgery”, and “elbow ulnar collateral ligament repair”. To avoid introducing search history bias, searches were each conducted on a newly installed Google Chrome web browser (Google Inc.) with no prior search history by one of the authors (J.T.S.) appeared on the page—surpassing the methods of previous studies which included 50-150 questions.^3^^,^^9^^,^^13^^,^^15^ Questions may present multiple times in the “People also ask” function, and questions which were exact matches were combined and a count of number of appearances was tabulated. The 3 search strings for both topics (UCL tear and UCL surgery) were combined for a total of 900 unique questions per topic, from which a list of the top 300 most frequently encountered questions for each topic (UCL tear and UCL surgery) was created.

For both topics, the collected questions and webpages were reviewed to determine if the “People also ask” question pertained to the group it was collected for (ie, a question and pertaining to ACL injury would be considered inconsistent with UCL tear topic). The same was done for the webpage associated with the “People also ask” question. Finally, the authors determined whether the “People also ask” question pertained to surgical details (ie, preoperative expectations, intraoperative or postoperative details) or were nonsurgical (ie, treatment decision-making, conservative treatment options, diagnostic modalities).

The “People also ask” question content was categorized using the Rothwell classification and subclassification system as in prior studies.^9^^,^^13^^,^^15^ The Rothwell classification includes 3 possible themes—fact, policy, or value. The subclassification includes specific activities, timeline of recovery, restrictions, technical details, cost, indications/management, risks/complications, pain, longevity, or evaluation of surgery. Table 1 provides descriptions of Rothwell classification and subclassification along with pertinent examples.

**Table I:** Description and examples of each type of Rothwell classification and subclassification.

Classification	Subclassification	Description/Example query
Fact		Ask whether something is true, and to what extent
	Specific Activities	“Can I drive after UCL surgery?”
	Timeline of Recovery	“What is the average recovery time after UCL surgery?”
	Technical Details	“How long does a UCL surgery take?”
	Restrictions	“What can you not do after UCL surgery?”
	Cost	“How much does UCL surgery cost?”
Policy		Ask whether a certain course of action should be taken to solve a problem
	Indications/Management	“What happens if a torn UCL goes untreated?”
	Risks/Complications	“Can you wait too long for UCL surgery?”
Value		Ask for evaluation of an idea, object, or event
	Pain	“Why is UCL surgery so painful?”
	Longevity	“How long does a UCL surgery last?”
	Evaluation of Surgery	“Is UCL surgery worth it?”

*UCL*, ulnar collateral ligament.

Each associated webpage was visited, and the source of information was categorized based on website host into academic, commercial, journal, government, legal, medical information, medical practice, nonmedical media, or single surgeon practice similar to prior investigations.^9^

Each webpage was scored for academic source reporting quality on a 4-point scale using the *Journal of the American Medical Association* (JAMA) benchmark criteria, which include points for authorship, attribution, currency, and disclosure.^15^^,^^16^ To get credit for authorship, the page must include clearly identifiable authors and contributors with affiliations and relevant credentials present. Attribution must have references and sources clearly listed with any copyright information disclosed. Currency implies clearly identifiable posting date of any content as well as date of any revisions. Finally, disclosures must clearly indicate website ownership along with any sponsorship, advertising, underwriting, and financial support.

All data categorization was performed by 2 independent authors (C.C.M. and B.R.U.). Discrepancies between authors were reviewed by a third author (A.J.H.) who functioned as a final tiebreaker.

### Statistical analysis

Cohen’s kappa coefficient was used to evaluate interobserver reliability of Rothwell classification, Rothwell subclassification, JAMA criteria, and website classification. Pearson’s chi-square test and Student’s *t*-test were used to evaluate the results for significance. Statistical significance was set to *P* values < .05.

## Results

The top 300 most frequently encountered “People also ask” questions for both UCL tear and UCL surgery topics were compiled and analyzed, for a total of 600 unique questions. The most common questions by Rothwell classification were fact for the UCL surgery cohort (57%) compared to 38% in the UCL tear cohort (*P* < .001). The most common classification in the UCL tear cohort was policy (55%) compared to 32% in the UCL surgery cohort (*P* < .001). The most common subcategories of Rothwell classification were indications/management in the UCL tear cohort (46%) compared to 25% in the UCL surgery cohort (*P* < .001). The most common subcategory for the UCL surgery cohort was tied between technical details (25%) and indications/management (25%). Questions were more likely to pertain to specific activities in the UCL surgery cohort (13%) compared to the UCL tear cohort (3%; *P* < .001). The UCL surgery cohort was also more likely to have questions about timeline of recovery (15%) compared to the UCL tear cohort (9%; *P* = .024). These results are shown in Table 2.

**Table II:** Rothwell subclassification counts and percentages comparing UCL tear and UCL surgery groups.

Rothwell classification	UCL tear (n = 300%)	UCL surgery (n = 300%)	*P* value
Fact	113 (38)	172 (57)	<.001∗
Timeline of recovery	27 (9)	45 (15)	.024∗
Specific activities	9 (3)	39 (13)	<.001∗
Technical details	71 (24)	75 (25)	.704
Restrictions	5 (2)	11 (4)	.128
Cost	1 (0)	2 (1)	.563
Policy	164 (55)	97 (32)	<.001∗
Indications/Management	137 (46)	75 (25)	<.001∗
Risks/Complications	27 (9)	22 (7)	.456
Value	23 (8)	31 (11)	.254
Pain	14 (5)	11 (4)	.540
Evaluation of surgery	8 (3)	15 (5)	.137
Longevity	1 (0)	5 (2)	.101

*UCL*, ulnar collateral ligament.

∗ Denotes statistical significance at a significance level < .05.

The most common webpage classifications encountered were academic in both cohorts, although the proportion was significantly higher in the UCL tear group (39%) compared to the UCL surgery group (29%; *P* = .008). The second most common webpage was medical practice (24% UCL tear, 26% UCL surgery; *P* = .510). The UCL surgery cohort trended toward having a higher proportion of single-surgeon personal websites, although this did not reach statistical significance (10% vs. 6%, *P* = .066). These results are illustrated in Table 3.

**Table III:** Website classification counts and percentages for UCL tear and surgery groups with associated JAMA scores.

Website classification	UCL tear (n = 300%)	UCL surgery (n = 300%)	*P* value	Mean JAMA score (±SD)
Academic (n = 205)	118 (39)	87 (29)	.008∗	0.6 ± 1.0
Commercial (n = 42)	19 (6)	23 (8)	.522	1.0 ± 1.0
Government (n = 60)	29 (10)	31 (10)	.785	2.8 ± 1.1
Journal (n = 25)	10 (3)	15 (5)	.307	3.4 ± 0.7
Legal (n = 3)	1 (0)	2 (1)	.563	0 ± 0
Medical Info Site (n = 60)	31 (10)	29 (10)	.785	2.5 ± 1.0
Medical Practice (n = 151)	72 (24)	79 (26)	.510	0.5 ± 0.8
Nonmedical Media Site (n = 8)	3 (1)	5 (2)	.477	1.5 ± 0.5
Single Surgeon Personal (n = 46)	17 (6)	29 (10)	.066	0.6 ± 0.8

*JAMA*, Journal of the American Medical Association; *SD*, standard deviation; *UCL*, ulnar collateral ligament.

∗ Denotes statistical significance at a significance level < .05.

The mean JAMA score for all webpages was 1.2 ± 1.3. Journal webpages had the highest average JAMA score (3.4 ± 0.7), whereas legal websites had the lowest average JAMA score (0 ± 0). Academic webpages had an average JAMA score of 0.6 ± 1.0 and medical practice webpages averaged 0.5 ± 0.8. The analysis for all webpage types is presented in Table 3.

The most frequently encountered “People also ask” question in the UCL tear search string was tied between “how long does it take to heal a torn UCL?” and “what is nonsurgical treatment for the UCL?” (2.7% frequency each). The most encountered search in the UCL surgery cohort was “can you retear your UCL after surgery?” at 3.0% frequency. The top 5 most frequently encountered searches for both cohorts are shown in Table 4.

**Table IV:** Most frequently encountered queries for UCL tear and UCL surgery groups.

	Count	Frequency %
UCL tear (n = 300)		
How long does it take to heal a torn UCL?	8	2.7
What is nonsurgical treatment for the UCL?	8	2.7
Will an MRI show a UCL tear?	7	2.3
What is the fastest way to heal a torn ligament in the elbow?	7	2.3
Can you rehab a torn UCL?	7	2.3
UCL surgery (n = 300)		
Can you retear your UCL after surgery?	9	3.0
How long is recovery from UCL repair?	8	2.7
What is the success rate of UCL reconstruction?	7	2.3
How long after UCL surgery can you drive?	6	2.0
Why is Tommy John surgery so common?	6	2.0

*UCL*, ulnar collateral ligament; *MRI*, magnetic resonance imaging.

From the search strings used in this study, 24% of the combined 600 populated “People also ask” questions explicitly mentioned the UCL in their question, for example, a string such as “what are the symptoms of a torn ulnar collateral ligament?”. From the associated webpages of the 600 questions, 30% of these contained information pertaining specifically to the UCL. Of the UCL tear cohort, 17% of questions pertained to surgical factors, including preoperative, intraoperative, and postoperative information. The UCL surgery cohort showed 56% of links contained surgical information.

Cohen’s kappa coefficient for inter-rater reliability was good for Rothwell classification (0.70) as well as subclassification (0.72), and very good for JAMA criteria (0.84) and website classification (0.85).

## Discussion

This study defines the most frequently searched online questions on the topic of both UCL tears and UCL surgery. The most searched question on UCL tears is, “How long does it take to heal a torn UCL?”. The most searched question on UCL surgery is, “Can you retear your UCL after surgery?”. The most common Rothwell classification for the UCL tear group is policy (55%), with most questions pertaining to the subcategory of indications/management (46%). The most common Rothwell classification for the UCL surgery cohort is fact (57%), with questions in the subcategory of technical details being the most common (25%). In both cohorts, academic websites were the most frequent website type encountered, although these were significantly more common in the UCL tear group compared to the surgery. Of the websites encountered, journal websites had the highest average JAMA score, while legal websites had the lowest (0 ± 0). Overall, only one-third of websites encountered contained information pertaining specifically to the UCL.

The most common questions in the UCL tear cohort pertained to policy. More specifically, patients are turning to the Internet for answers on management of UCL tears and indications for surgery. The most common questions were “How long does it take to heal a torn UCL?” and “What is nonsurgical treatment for the UCL?”. This demonstrates that patients appear to be most interested in learning what modalities are used in nonoperative management and their recovery timeline after a UCL injury. When counseling patients with recently diagnosed tears, it is important for surgeons to focus patient education on the treatment options available and what patients can expect during their rehabilitation process, particularly time to full activity.

Most questions in the UCL surgery group were fact-based, with the most common subcategory of involving the technical details of surgery. This subcategory is followed closely by questions on timeline for recovery, specific activity restrictions, and indications/management. While patients searching about UCL tears are more focused on learning what treatment options are available, those inquiring about UCL surgery are interested in learning details of surgical management. Prior to surgery, we recommend that surgeons engage their patients in a thorough discussion that includes a description of the proposed operation and details on what to expect in the perioperative period to avoid confusion and misinformation. The importance of setting appropriate preoperative expectations has been previously found to correlate with improved postoperative outcomes and patient satisfaction in orthopedic procedures, highlighting just how important these patient questions are.^12^^,^^18^^,^^19^

Academic websites were the most frequently encountered in both cohorts, although they were more commonly encountered in the UCL tear group. The second most common category of webpage was medical practice. Unfortunately, websites encountered in both of these categories had very poor academic credibility according to the JAMA benchmark criteria. Online journals had the highest JAMA score; however, information on these sites can be complex and difficult to comprehend for those with limited medical knowledge. Most websites encountered in this study failed to list authors of their sources and their credentials, cite appropriate information, or include dates when information was updated. This finding is consistent with previous studies which have found that the online medical information can be misleading, fail to report on sources, and often lack a basis in scientific data.^4^ Within the field of orthopedics, several studies have demonstrated the poor quality of online information.^3^^,^^14^^,^^17^ Yu et al recently demonstrated that information found on Youtube regarding UCL injuries in highly variable and often inaccurate, which has been corroborated in a systematic review by Desai et al.^5^^,^^20^ It is imperative that surgeons appropriately educate their patients to ensure they are not subjected to inaccurate and potentially harmful information on the Internet.^10^ Furthermore, orthopedic providers and those involved in patient education have a responsibility to publish accurate and reliable information when publishing online resources for patients.

Our analysis found that webpages provided through the “People also ask” function pertained to the UCL only one-third of the time despite all original search strings referencing the UCL by name. This finding highlights the utility of the search suggestions to keep patients engaged by providing related information. It also illustrates clearly how patients can become misinformed with only a few clicks, as they happen upon links with references to similar but separate pathology or anatomy and unrelated treatment regimens. We recommend surgeons educate their patients on the relative frequency of links pertaining to separate pathology and empower them to confirm the information they are reading is actually related to their injury.

The most significant limitation of this study is the use of the Google “People also ask” function. This function uses machine learning to predict what an individual user may search next based on previously collected data from others searching for information on the same topic. This prediction is heavily influenced by that specific individual’s prior search history. This means that the algorithm could suggest a different list of questions between 2 users. To mitigate this variability, we included a larger number of questions than prior studies and performed all searches on a cleanly installed web browser with no previously conducted searches. Our search methodology was devised to mimic the information a typical patient would find via Google search; however, we acknowledge that the actual content seen by a patient may vary subtly based on an individual user’s prior search history. These questions do not come directly from searches of orthopedic patients and it is not possible for us to confirm the identity of those searching these questions. We cannot confirm if these questions are being asked preoperatively or postoperatively by patients. However, the utilization of the “People also ask” function allows us to find the questions being asked by patients in the context of online anonymity. These searches were conducted in early 2023 and we limited the search country to only the United States. It is possible that our sample of questions is not representative of current trends as more information becomes available on the Internet and it may not be representative of a sample of patients outside of the United States. Finally, the JAMA benchmark criteria is a flawed measure for assessing the accuracy and reliability of online information, as mentioned in the original manuscript.^16^ Although it is important for websites to include authorship information, proper citations, accurate dates, and appropriate disclosures, this is merely a proxy of the validity of information provided.

## Conclusion

The most common online patient questions pertaining to UCL surgery pertain to technical details and indications, while nearly half of questions related to UCL tears encompass injury management. Webpages suggested by online search engines contain information specific to UCL tears and surgery only one-third of the time. The quality of most webpages provided to patients is poor, with minimal source transparency.

## Disclaimers

Funding: No funding was disclosed by the authors.

Conflicts of interest: Dr. Chahla reports being a paid consultant from Arthrex, Inc, CONMED Linvatec, Ossur, and Smith & Nephew, being a paid presenter or speaker for Smith & Nephew, and a Board of Committee member of the American Orthopaedic Society for Sports Medicine, Arthroscopy Association of North America, and the International Society of Arthroscopy, Knee Surgery, and Orthopaedic Sports Medicine. Dr. Verma reports research support from Arthrex, Breg, Ossur, Smith & Nephew, and Stryker, IP royalties from Arthrex, Smith & Nephew, and Stryker, and he is a board or committee member for the 10.13039/100011549American Orthopaedic Society for Sports Medicine, 10.13039/100013615American Shoulder and Elbow Surgeons, and 10.13039/100008542Arthroscopy Association of North America. He is also on the Editorial or Governing board of SLACK incorporated. The other authors, their immediate families, and any research foundation with which they are affiliated have not received any financial payments or other benefits from any commercial entity related to the subject of this article.
